# Supplementary material for: Effects of Lactococcus cremoris PS133 in 5-Hydroxytryptophan-Induced Irritable Bowel Syndrome Model Rats
Source: Int J Mol Sci. 2025 Mar 10;26(6):2464. doi: 10.3390/ijms26062464 (PMC11942017; doi:10.3390/ijms26062464)
Supplement: Supplementary file 1 [file ijms-26-02464-s001.zip › ijms-3487629-supplementary.pdf]

Supplementary Figure S1

(A) (B)

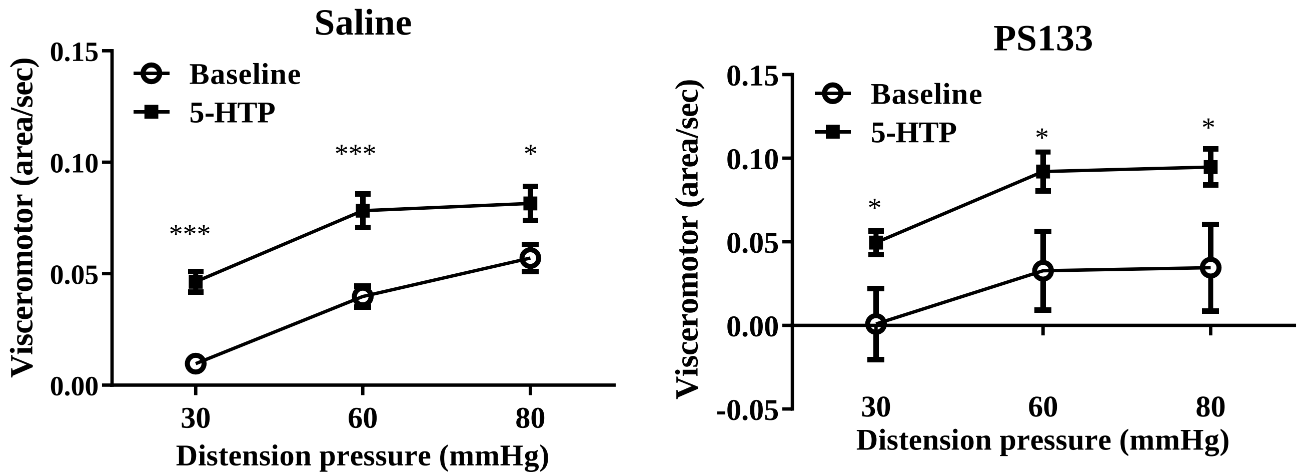

Figure S1. Effect of *L. cremoris* PS133 on 5-HTP-induced VH on experimental day 14. The visceromotor responses were recorded 30 min before (baseline) and after the injection of 5-HTP to (A) Saline group and (B) PS133 group. *$p < 0.05$; ***$p < 0.001$, compared with the baseline by repeated two-way ANOVA with Bonferroni correction.

Supplementary Figure S2

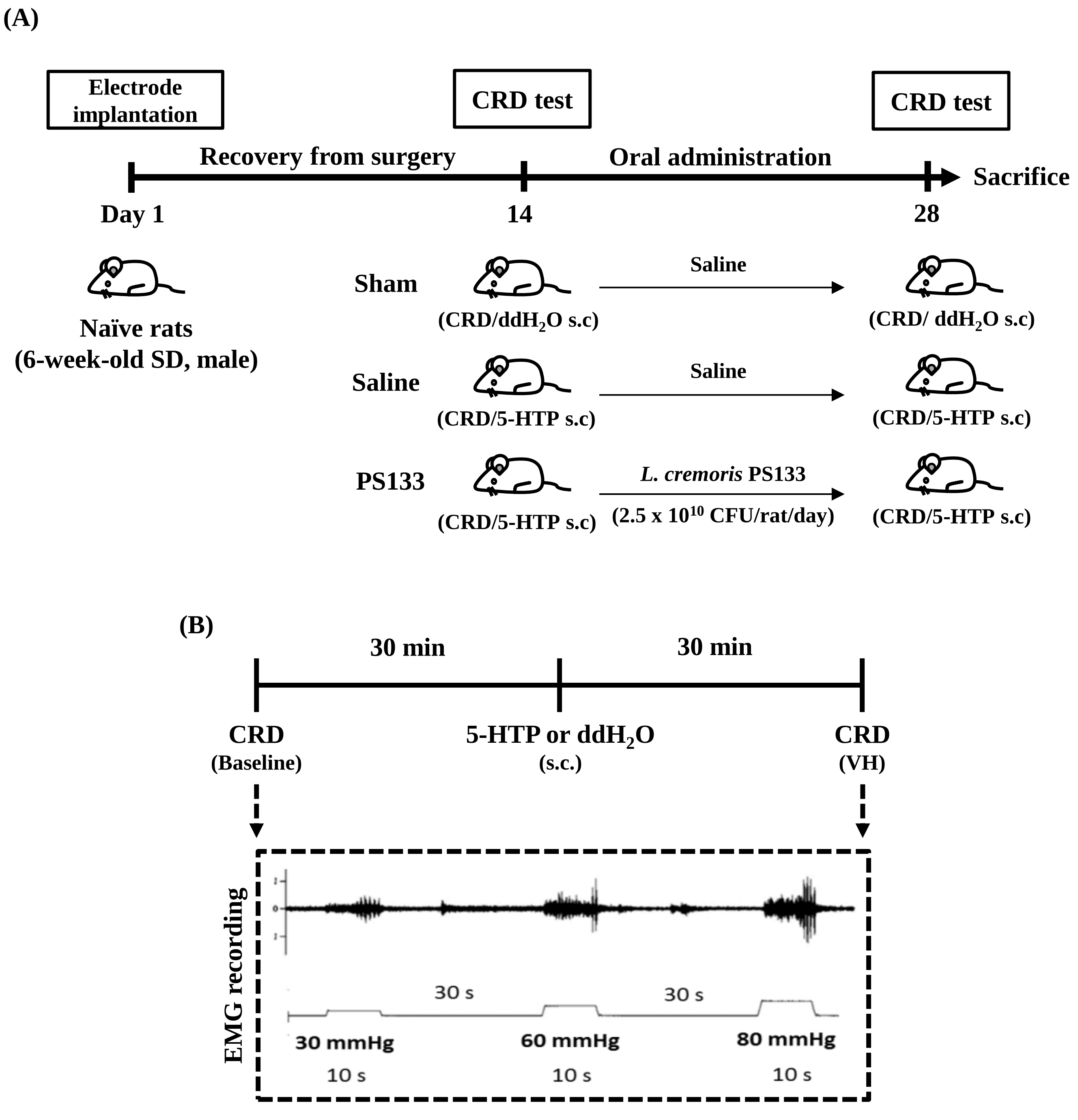

Figure S2. Scheme of the animal experimental design. Rats were assigned to four groups randomly (Naïve group, Sham group, Saline group, and PS133 group, n = 32). After electrode implantation and recovery (Day 1 to Day 14), rats were administered *L. cremoris* PS133 or saline for two weeks (Day 14 to Day 28). Visceral hypersensitivity (VH) was induced by subscutaneously injecting 5-HTP on Day 14 and Day 28. (B) Colorectal distension (CRD) was performed on the colon with inflating ballon to desired pressure (30, 60, or 80 mmHg) for 10-second intervals with 30-second intervals between distensions. The visceromotor responses were recorded by electromyography (EMG). In the end, the rats were sacrificed immediately after the last CRD test.

Supplementary Figure S3

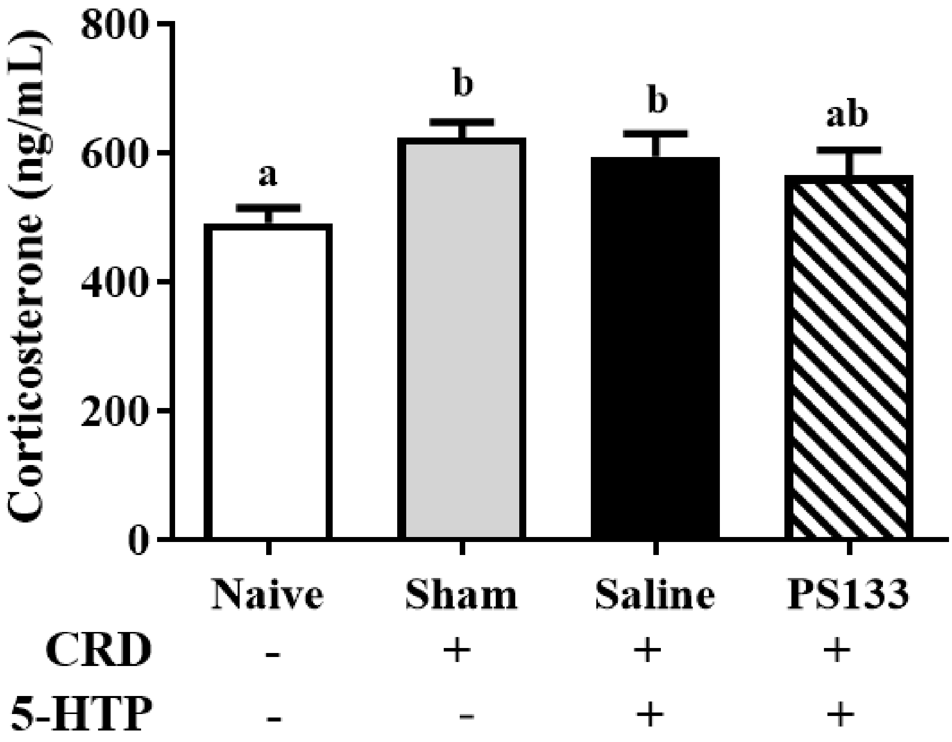

Figure S3. Measurements of corticosterone concentrations in serum. Corticosterone level extracted from serum of each group was shown. Data were expressed as mean ± S.E.M and analyzed by two-way ANOVA with Tukey's post hoc test.

Supplementary Figure S4

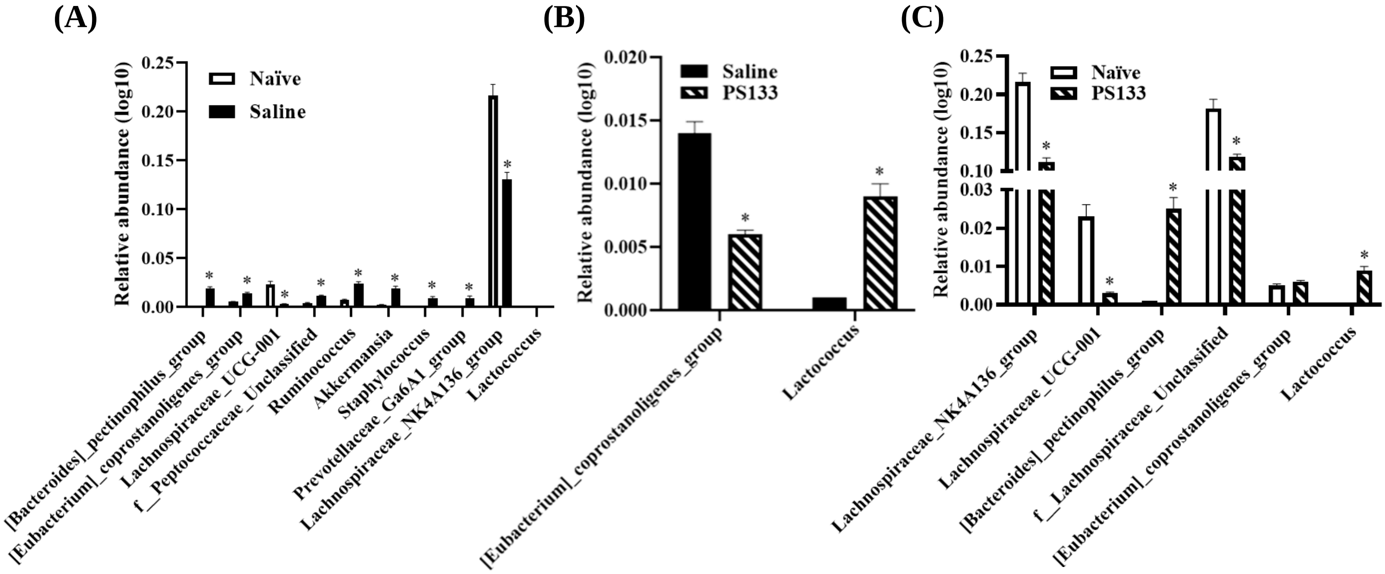

Figure S4. Analysis of the relative abundance of bacteria at the genus level. Comparison of relative abundances of bacteria between (A) Naïve and Saline; (B) Saline and PS133; and (C) Naïve and PS133 groups. Data was expressed as the mean ± SD. * means $p < 0.05$, two-tailed Student *t*-test.
